# Introduction: Novel Frontiers in Cancer Metastasis

**DOI:** 10.1007/s10585-022-10151-0

**Published:** 2022-02-22

**Authors:** Stanley P. Leong, Jonathan S. Zager

**Affiliations:** 1 California Pacific Medical Center and Research Institute, San Francisco, CA USA; 2 University of California San Francisco School of Medicine, San Francisco, CA USA; 3 Department of Cutaneous Oncology, Moffitt Cancer Center, Tampa, FL USA; 4 University of South Florida Morsani College of Medicine, Tampa, USA

**Keywords:** Cancer heterogeneity, Cancer mutations, Cancer microenvrionment and metastasis

The 8th International Cancer Metastasis Congress through the Lymphovascular System: Biology & Treatment, was held from October 25–27, 2019 in San Francisco, California. This international conference was inaugurated in 2005 and has been successfully held biennially with several publications summarizing the biology and treatment of cancer metastasis. The last conference in 2017 was summarized in a special issue by Clinical and Experimental Metastasis [1]. The 8th International Cancer Metastasis Congress in October 2019 in San Francisco [2] addressed four major themes including cancer heterogeneity due to mutation [3, 4] with association of formation of neoantigens [5, 6], cancer progression relating to the cancer microenvironment and the mechanisms of metastasis through the lymphatic versus blood vessels and cancer therapy. These major topics are covered in 23 review articles being published online and they have been incorporated into this Special Issue: Novel Frontiers in Cancer Metastasis [7].

Cancer heterogeneity is discussed in the context of Breast Cancer Disparities in Outcomes; Unmasking Biological Determinates Associated with Racial and Genetic Diversity [8] and Cancer Heterogeneity and Metastasis: Life at the Edge [9].

The diversity of cancer biomarkers further validate that cancer is heterogeneous as represented by several review articles on Detection of Cancer Metastasis: Past, Present and Future [10]; Sentinel Lymph Node Biopsy of Melanoma Beyond Histologic Factors [11]; Detection of Melanoma, Breast Cancer and Head and Neck Squamous Cell Cancer Sentinel Lymph Nodes by Tc-99 m Tilmanocept [12]; Cancer Neoantigens as Potential Targets for Immunotherapy [6]; and Current Status of Gastrointestinal Tract Cancer Brain Metastasis and the Use of Blood-based Cancer Biomarker Biopsy [13].

Based on the heterogeneous array of biomarkers, personalized cancer therapy is developed against specific biomarkers for more accurate targeted therapy as represented by the two review articles on Precision Oncology for Breast Cancer through Clinical Trials [14] and Bringing Precision Oncology to Cellular Resolution with Single-Cell Genomics [15].

The role of cancer microenvironment exerts a selective force akin to Darwin’s natural selection [16–18] in the modulation and forging of more aggressive cancer clones to metastasize as reflected in the review article on Cancer Microenvironment and Genomics: Evolution in Process [19].

Cancer clones may escape the primary site and spread initially to the sentinel lymph node as summarized in the Donald L. Morton’s Memorial Lecture: The Legacy of Donald Morton: Past, Present and Future [20] to suggest that sentinel lymph nodes may serve as a major gateway or conduit for cancer metastasis. All the patients with a negative sentinel lymph node biopsy are spared of a radical regional lymph node dissection. The unique role of sentinel lymph nodes in the management of colorectal cancer is addressed by the review article on the Role of Sentinel Lymph Node Mapping in Colon Cancer: Detection of Micrometastasis, Effect on Survival, and Driver of a Paradigm Shift in Extent of Colon Cancer [21].

The molecular mechanism of cancer metastasis through the lymphatic versus blood vessels is still not well understood. An attempt has been made to summarize what we know to date relating to the mechanisms of cancer metastasis in the following review articles on Mechanisms of Breast Cancer Metastasis [22]; The Lymphatic System and Sentinel Lymph Nodes: Conduit for Cancer Metastasis [23]; and Molecular Mechanisms of Cancer Metastasis via the Lymphatic versus the Blood Vessels [24].

The role of lymph node dissection and systemic treatment for a positive sentinel lymph node biopsy are discussed in depth using the model of melanoma, entitled Current Management of Melanoma Patients with Nodal Metastases [25]. In-transit metastasis within the lymphatic vessels is unique in melanoma and it is addressed in The treatment of In-Transit Metastatic Melanoma [26].

Additional novel imaging of cancer is illustrated by Emerging Photoacoustic and Thermoacoustic Imaging Techniques for Detecting Primary and Metastatic Cancer and Guiding Therapy [27]. Novel radio-imaging and radiotherapy are discussed in Introduction to Novel Developments in Radio-Imaging and Radiotherapy [28].

Further, innovations in radiotherapy are described in Innovations in Radiotherapy and Advances in Immunotherapy for the Treatment of Brain Metastases [29]. Check point inhibition therapy is discussed in Immunotherapy of Cancer in the Era of Checkpoint Inhibitor [30].

Secondary Lymphedema Related to Cancer Treatment by Surgery or Radiotherapy is an important topic relating to the treatment complication of cancer [31].

The biological events of cancer development with mutation and cancer evolution as well as the molecular mechanisms of cancer metastasis may be more accurately correlated and defined by the adoption of artificial intelligence as summarized in the review article on Empowering Study of Breast Cancer Data with Application of Artificial Intelligence Technology: Promises, Challenges, and Use Cases [32].

This Special Issue: Novel Frontiers in Cancer Metastasis emphasizes that cancer is a heterogeneous disease resulting from genetic mutations or epigenetic changes [33]. Such heterogeneity is characterized by the development of different clones within the primary cancer cell population. Akin to Darwinian “natural selection” [18], the cancer microenvironment including host’s immune responses exerts a selective force to promote the development of aggressive clones to invade and metastasize from the primary site to the distant sites, in accordance to Paget’s seed and soil hypothesis for cancer spread [34], through the lymphatic or blood vessels. Cancer heterogeneity may be present intratumorally, in various sites of metastasis or among patients. Thus, personalized cancer therapy is being developed to treat cancer based on its unique characteristics in relation to each patient. Future molecular and genetic studies with the aid of artificial intelligence may further help us to understand cancer heterogeneity, progression and metastasis with increased precision, thus, allowing the development of more effective therapeutic strategy to control cancer growth.

## Data Availability

Data sharing not applicable to this article as no datasets were generated or analyzed during the current commentary.
